# Differential Treatment Response Between Hypochondriasis With and Without Prominent Somatic Symptoms

**DOI:** 10.3389/fpsyt.2021.691703

**Published:** 2021-11-08

**Authors:** Brian A. Fallon, Cale Basaraba, Martina Pavlicova, David K. Ahern, Arthur J. Barsky

**Affiliations:** ^1^Department of Psychiatry, Columbia University, New York, NY, United States; ^2^Department of Psychiatry, New York State Psychiatric Institute, New York, NY, United States; ^3^Department of Biostatistics, Mailman School of Public Health, Columbia University, New York, NY, United States; ^4^Department of Psychiatry, Brigham and Women's Hospital, Harvard University, Boston, MA, United States

**Keywords:** hypochondriasis, illness anxiety disorder, somatic symptom disorder, cognitive behavioral therapy, fluoxetine, treatment response

## Abstract

**Background:** Health anxiety may exist with or without prominent somatic symptoms, but the impact of somatic symptoms on treatment response is unclear. The study objective was to examine this question further as symptom burden may impact choice of type of treatment.

**Methods:** This exploratory study used a unique database from a prior trial of 193 individuals with DSM-IV hypochondriasis who had been randomly assigned to either cognitive behavioral therapy, fluoxetine, combined therapy, or placebo. Two subgroups were newly defined—no/low somatic burden (*n* = 42) and prominent somatic burden (*n* = 151). Response was defined by ≥30% improvement in hypochondriasis.

**Results:** Among high somatic hypochondriacal participants, compared to placebo, the odds of being a responder were significantly greater among those who received fluoxetine, either alone (OR = 4.46; 95% CI: 1.38, 14.41) or with cognitive behavioral therapy (OR = 3.56; 95% CI: 1.19, 10.68); the estimated odds were not significantly different for those receiving cognitive behavioral therapy alone (OR = 1.81; 95% CI: 0.59, 5.54). In contrast, among low somatic hypochondriacal participants, compared to placebo, the observed odds of being a responder were similar in magnitude and direction for those who received cognitive behavioral therapy, either alone (OR = 3.00; 95% CI: 0.38, 23.68) or in combination with fluoxetine (OR = 3.60; 95% CI: 0.62, 21.03), compared to the odds for those receiving fluoxetine alone (OR = 0.90; 95% CI: 0.14, 5.65). High somatic hypochondriacal individuals assigned to any fluoxetine group had significantly greater odds of being a responder than those who had not received fluoxetine (OR = 2.70; 95% CI: 1.33, 5.48). Low somatic hypochondriacal individuals assigned to any cognitive behavioral therapy group had significantly greater odds of being a responder than those who had not received cognitive behavioral therapy (OR = 8.03; 95% CI: 1.41, 45.67).

**Conclusion:** These findings indicate that somatic symptom burden may be important in guiding treatment selection among individuals with marked health anxiety, as hypochondriacal individuals with high somatic burden responded more often to fluoxetine while those with low somatic burden responded more often to cognitive behavioral therapy. Systematic replication with larger studies is needed.

## Introduction

Hypochondriasis is a prevalent and disabling disorder for which pharmacotherapy and cognitive behavioral therapy have each been shown to reduce hypochondriacal symptoms (1). Among individuals with hypochondriasis, ~75% have prominent somatic symptoms while 25% have mild or no somatic symptoms (2). In DSM-5, the former group with prominent somatic symptoms would be diagnosed with Somatic Symptom Disorder (SSD), while the latter group without prominent somatic symptoms would be diagnosed with Illness Anxiety Disorder (IAD). While knowledge about somatic symptom burden is important for diagnostic assessment in DSM-5, is it important for treatment selection?

The present exploratory study aims to address this question by comparing treatment response among individuals with hypochondriasis with and without prominent somatic symptoms. We use data collected during a prior large randomized controlled clinical trial of pharmacotherapy vs. cognitive behavioral therapy (CBT) for DSM-IV hypochondriasis (3). That study confirmed the predicted pattern of efficacy: joint treatment with CBT and fluoxetine had higher responder rates than individual therapy alone which in turn had higher rates than placebo. That study also demonstrated that both fluoxetine and CBT were well-tolerated, as no difference was found across the 4 treatment groups in the rates of study drop-outs or in the rates of treatment-emergent adverse effects. Although somatic symptoms were assessed, their contribution to treatment response was not examined. Because of the DSM-5's emphasis on the categorical importance of somatic symptoms, for this new exploratory analysis we reclassified the participants with DSM-IV hypochondriasis from our prior clinical trial into two subgroups—those with low somatic (HYP-LS) severity and those with high somatic (HYP-HS) severity. If a differential treatment response to psychotherapy or pharmacotherapy were found in the reduction of hypochondriacal symptoms within these two subgroups, this would suggest that somatic symptom burden may be a useful guide for clinicians in treatment selection to optimize treatment response.

## Materials and Methods

### Original Study Design and Procedure

In the original study (3), 193 participants meeting DSM-IV criteria for hypochondriasis were randomly assigned to one of 4 manualized treatments fluoxetine (FLX) plus medical management supportive therapy (MMST); placebo (PBO) plus MMST; cognitive-behavioral therapy (CBT); or cognitive-behavioral therapy plus fluoxetine (CBT/FLX). The FLX arm and the PBO arms of the study were triple-blinded for the clinician, participant, and independent evaluator (who conducted the H-YBOCS-M assessment). The CBT and the CBT/FLX arms were single-blinded in that only the independent evaluator was uninformed of the treatment allocation. The study was conducted at two sites in the United States (New York City and Boston), following identical design and procedure. Briefly, the manualized CBT consisted of 6 in-person 60 min weekly intensive in person sessions followed by 2 biweekly and then 3 monthly booster sessions. The treatment modules addressed psychoeducation about illness anxiety and the role of attention and context, reduction of bodily hypervigilance, reformulation of dysfunctional assumptions about symptom etiology, modification of confirmatory bias, and reduction of maladaptive sick role behaviors; exposure therapy was not a treatment component. The pharmacotherapy consisted of daily fluoxetine starting at 10 mg and increasing as needed and tolerated to 80 mg/day. Both the fluoxetine and placebo randomized groups received manualized medication management supportive therapy, matching the number of visits occurring in the CBT groups, but for a briefer timeframe of 20–30 min. Primary outcome was assessed at 6 months. Further details of the study methods, procedures, and study population can be found in a prior publication (3). The study was approved by the Institutional Review Boards at both institutions and registered at clinicaltrials.gov (NCT00339079). All participants provided written informed consent. Fluoxetine is an FDA approved medication for the treatment of major depressive disorder, obsessive compulsive disorder, panic disorder, and bulimia nervosa; fluoxetine has not received FDA approval for use in hypochondriasis.

### Participants

Participants were recruited from the community and were included if they had a primary diagnosis of DSM-IV hypochondriasis of at least “moderate” severity, were not taking psychotropic medications, and did not have a major comorbid psychiatric disorder (schizophrenia, schizoaffective disorder, delusional disorder, substance abuse or dependence disorder, or bipolar disorder). If another comorbid psychiatric disorder (e.g., major depression) was present, then hypochondriasis was judged to be the primary, predominant disorder. In addition, individuals with a co-morbid psychiatric disorder causing significant impairment in vocational or social role function were excluded.

### Measures

The expanded MINI International Neuropsychiatric Interview (4) was used to establish DSM-IV diagnosis. The severity of hypochondriacal symptoms was assessed using the H-YBOCS-M which is a semi-structured, clinician-administered instrument (5) as well as by the Whiteley Index of Hypochondriasis which is a 14-item, Likert-type self-report (6). Somatic symptoms were assessed using the Patient Health Questionnaire-15 (7)—a 15-item self-report measure that assesses somatic symptoms during the prior 2 weeks. Symptoms are rated on a 0–2 scale where 0 = “not bothered at all,” 1 = “bothered a little,” and 2 = “bothered a lot.” Mood was assessed using the following self-report measures: the Beck Depression Inventory-II (BDI-II) (8) and the State-Trait Anxiety Inventory (STAI) (9). Quality of life was assessed with the 16-item self-report Quality of Life Enjoyment and Satisfaction Questionnaire (Q-LES-Q) (10) which assesses respondent's enjoyment and satisfaction in daily life.

### Statistical Methods for Hypochondriasis With High vs. Low Somatic Burden

For this exploratory analysis using a pre-existing dataset, the hypochondriacal sample was divided into those with high somatic (HYP-HS) and low somatic (HYP-LS) symptom burden using the pre-treatment PHQ-15 self-report somatic symptom measure. The HYP-HS group was composed of those individuals who rated at least one somatic symptom as bothering them “a lot” (approximating the hypochondriacal subtype of DSM-somatic symptom disorder [SSD]). The HYP-LS group was composed of hypochondriacal individuals who did not rate any PHQ-15 somatic symptom as bothering them “a lot” (approximating the DSM-5 diagnosis of illness anxiety disorder [IAD]). This same operational approach to identify likely IAD and SSD among individuals with hypochondriasis has been used previously (11).

The primary outcome was the dichotomous composite variable of treatment response at 24 weeks, defined in this analysis by the dual requirement of achieving at least 30% improvement over pre-treatment scores on two measures of hypochondriasis (the self-report Whiteley Index) and the clinician-administered measure (H-YBOCS-M). The last observed scores were carried forward for those who dropped out before week 24.

To explore the effect of treatment group on responder status, HYP-HS and HYP-LS groups were examined separately using a three-step approach. First, the odds ratios for the association between treatment group (FLX, CBT, CBT+FLX) and responder status, compared to placebo (PBO), was calculated. In the larger HYP-HS group, a logistic regression model was used to estimate the odds ratio, adjusted by baseline BDI score. In the smaller HYP-LS group, with fewer than 10 observations in the CBT group, only observed odds ratios and 95% confidence intervals were calculated, since extensive modeling is inappropriate for small sample sizes. An odds ratio was obtained for each treatment group, quantifying the odds of being a responder for each treatment compared to the PBO group.

In the second step of this exploratory analysis, treatment groups that had very similar odds (in terms of magnitude and direction) of responder status (i.e., they performed similarly compared to PBO) were collapsed into one new combined group; this shrinkage and grouping method was first described by Tukey as an element of exploratory data analysis (12). In summary, if the odds of being a responder differed substantially between all treatment groups, no groups would be combined, and no additional analyses would be run. If the odds of being a responder were similar between some treatment groups, those would be collapsed into one group and the analysis would proceed to the third step—the odds of responder status would be estimated using the new combined groups. For both HYP-HS and HYP-LS groups, logistic regression models that were adjusted by baseline BDI score were used to estimate odds ratios.

To assess the robustness of our results, the same three-step approach was applied to a secondary treatment response outcome, which was defined as 40% improvement on both the Whiteley Index and on the H-YBOCS-M at 24 weeks compared to baseline for each subject.

Additionally, the fairly large size of the HYP-HS group allowed us to explore potential baseline moderators to assess whether the treatment was more effective for certain subjects. The moderators assessed were baseline Whiteley Score (dichotomized at median), baseline H-YBOCS-M score (dichotomized at median), and baseline BDI score (dichotomized at clinically relevant cutoffs: first at ≥ 20 for at least moderate and second ≥29 for severe). For each moderator we ran logistic regression with the moderator-by-new groups interaction term adjusted by baseline BDI. Due to the small sample size of the HYP-LS group, no moderation analyses were performed.

To evaluate the impact of the choice of cutoff used on the PHQ-15 that defined the HYP-HS and HYP-LS groups, a sensitivity analysis was conducted which varied the number of PHQ-15 items required to be endorsed at the “Bothered a lot” level.

Adherence to CBT and FLX was assessed using means as well as proportions and compared between groups using *t*-test and chi-square tests.

All statistical tests were performed at a two-tailed level of significance of 5%. All analyses were performed using SAS® 9.4.

## Results

Of the 193 participants, 151 (78.2%) met criteria for HYP-HS and 42 (21.8%) met criteria for HYP-LS. Table 1 compares the treatment groups on baseline characteristics. Across clinical measures, the HYP-HS group had consistently greater clinical severity and lower quality of life than the HYP-LS group.

**Table 1:** Demographic and Clinical characteristics.

	**HYP-LS (*****n*** **= 42)**				**HYP-HS (*****n*** **= 151)**				**Diff between groups**
	**Mean**	**SD**	***n***	**%**	**Mean**	**SD**	***n***	**%**	***p*-value^a^**
**Demographics**									
Male			24	57.1			61	40.4	0.079
Hispanic			4	9.5			18	11.9	0.875
Single			25	59.5			94	62.3	0.843
Age (years)	39.6	15.6			39.7	14.0			0.948
Education (years)	15.5	2.8			15.4	2.5			0.961
**Baseline Clinical Characteristics**									
OCD			6	14.3			19^b^	12.6	0.797
Major depression			8	19.0			55^c^	36.4	0.040
Panic Disorder			2	4.8			25^c^	16.6	0.048
Whiteley Index	44.9	11.5			50.6	8.7			0.001
H-YBOCS-M	31.2	11.8			36.7	10.7			0.005
PHQ-15	5.9	2.7			11.4	4.0			<0.001
BDI-II	10.2	7.9			18.4^d^	11.5			<0.001
STAI	42.8	13.6			54.0^b^	12.7			<0.001
Q-LES-Q	49.5	9.3			42.1	9.8			<0.001

a *Differences between groups are assessed using t-tests for continuous measures and chi-square tests (or Fisher Exact tests) for categorical measures;*

b *Missing one observation;*

c *Missing two observations;*

d *Missing three observations.*

*HYP-LS, Hypochondriasis-Low Somatic; HYP-HS, Hypochondriasis-High Somatic; OCD, Obsessive Compulsive Disorder; H-YBOCS-M, Hypochondriasis-Yale Brown Obsessive Compulsive Scale-Modified; PHQ-15, Patient Health Questionnaire-15; BDI-II, Beck Depression Inventory-II; STAI, State-Trait Anxiety Index; Q-LES-Q, Quality of Life Enjoyment and Satisfaction Questionnaire*.

### Hypochondriacal Participants With High Somatic Burden

Of the 151 HYP-HS participants, 53 (35.0%) individuals met criteria for treatment response, with the highest proportion of responders found in the FLX treatment group (46.9%) followed by the CBT+FLX group (45.2%) (Table 2, HYP-HS Observed). As shown in Table 2 HYP-HS model estimates, the model-estimated odds of being a responder in the two groups that received medication were significantly higher when compared to PBO (FLX OR = 4.46; 95% CI: 1.38, 14.41; and CBT+FLX OR = 3.56; 95% CI: 1.19, 10.68), while adjusting for baseline BDI score.

**Table 2:** Observed Responder Status and Model Estimates by randomly assigned treatment group in HYP-HS and HYP-LS patients.

**HYP-HS**		**PBO**	**CBT**	**FLX**	**CBT+FLX**	**Total**
		**(*n* = 31)**	**(*n* = 46)**	**(*n* = 32)**	**(*n* = 42)**	**(*n* = 151)**
	**Observed**					
	Responder (30% Improvement), *n (%)*	6 (19.4%)	13 (28.3%)	15 (46.9%)	19 (45.2%)	53 (35.0%)
	Responder (40% Improvement), *n (%)*	4 (12.9%)	9 (19.6%)	14 (43.8%)	11 (26.2%)	38 (25.2%)
	**Model Estimated Odds Ratios** ^a^					
	Responder (30% Improvement), *OR (95% CI)*	(ref)	1.81 (0.59, 5.54)	4.46 (1.38, 14.41)	3.56 (1.19, 10.68)	
**HYP-LS**		**PBO**	**CBT**	**FLX**	**CBT+FLX**	**Total**
		**(*****n*** **= 12)**	**(*****n*** **= 6)**	**(*****n*** **= 13)**	**(*****n*** **= 11)**	**(*****n*** **= 42)**
	**Observed**					
	Responder (30% Improvement), *n (%)*	3 (25.0%)	3 (50.0%)	3 (23.1%)	6 (54.5%)	15 (35.7%)
	Responder (40% Improvement), *n (%)*	2 (16.7%)	3 (50.0%)	3 (23.1%)	5 (45.5%)	13 (31.0%)
	**Observed Odds Ratios**					
	Responder (30% Improvement), *OR (95% CI)*	(ref)	3.00 (0.38, 23.68)	0.90 (0.14, 5.65)	3.60 (0.62, 21.03)	

a *Adjusted for baseline BDI score*.

*HYP-LS, Hypochondriasis-Low Somatic; HYP-HS, Hypochondriasis-High Somatic*.

Since the odds of being a responder were similar in magnitude and direction in the two groups that received medication, in the second exploratory step, the FLX and the CBT+FLX groups were combined into one larger group labeled FLX+. The model-estimated odds of responder status in the CBT group were not significantly different from PBO and therefore CBT and PBO groups were combined into a new larger group termed FLX- for additional analyses.

**Figure 1:**
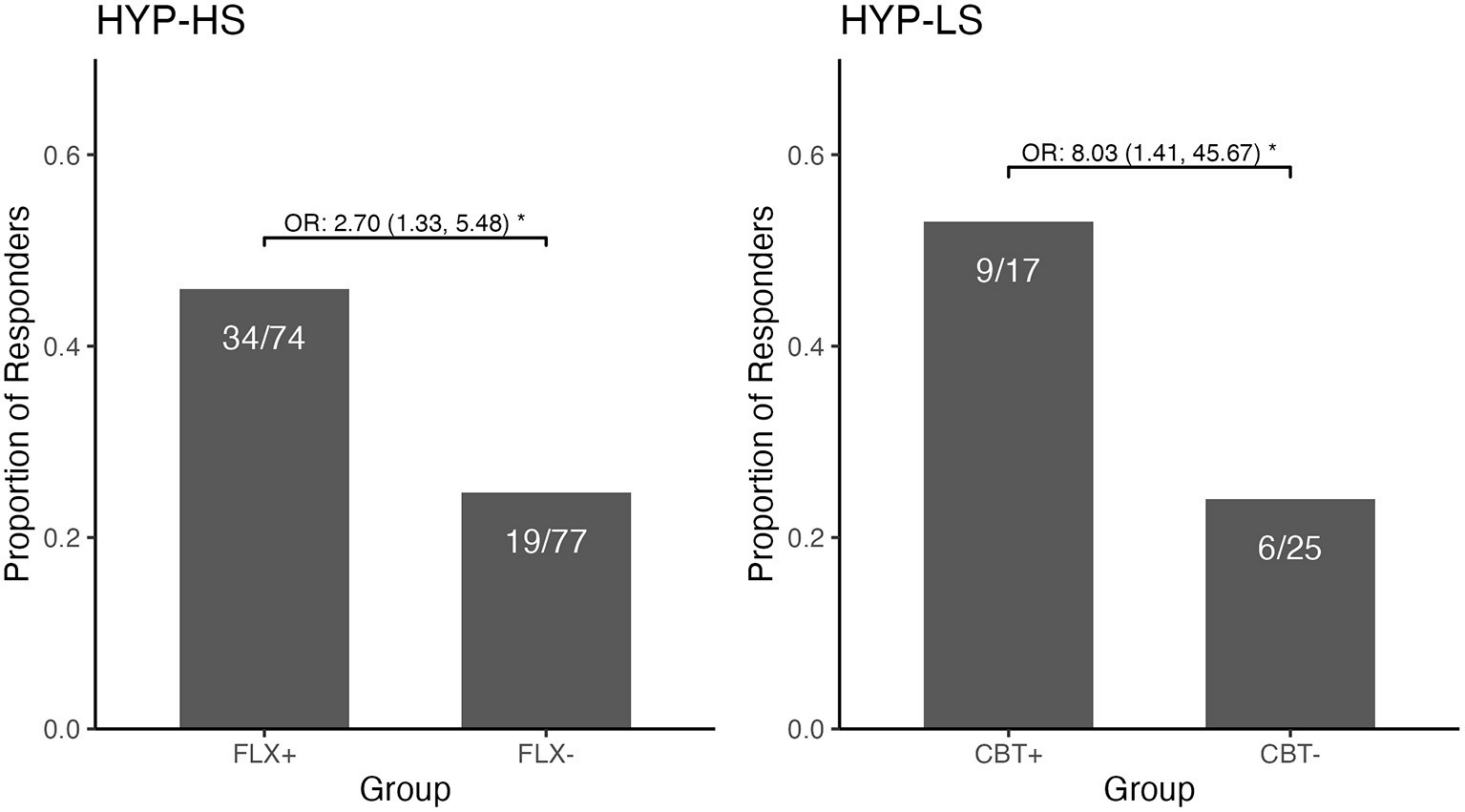
Observed Responder Status and Odds Ratio for the Combined Fluoxetine Groups and the Combined CBT Groups among those with Hypochondriasis—High Somatic and those with Hypochondriasis—Low Somatic. The figure demonstrates two contrasting response profiles. Among the HYP-High Somatic participants, there was a significantly greater likelihood of being a responder if in the combined fluoxetine group than if in the combined group without fluoxetine. Conversely, among the HYP-Low Somatic participants, there was a significantly greater likelihood of being a responder if in the combined CBT group than if in the combined group not receiving CBT. *p < 0.05.

In the final step we compared the two new combined groups: those who had received fluoxetine (FLX+) and those who had not (FLX-). In the FLX+ combined group, 34/74 (45.9%) participants were rated as treatment responders. In the FLX- combined group, 19/77 (24.7%) participants were responders (see Figure 1 and Table 3, Observed). The model-estimated odds of being a responder were significantly different between the FLX+ and FLX- combined groups (*p* = 0.006). Participants in the FLX+ combined group had 2.70 times (95% CI: 1.33, 5.48) the odds of being a responder compared to participants in the FLX- combined group, while adjusting for baseline BDI score (see Table 3, Model Estimated Odds Ratios).

**Table 3:** Observed Responder Status and Model Estimates by combined FLX+ and combined FLX- groups in HYP-HS participants and by combined CBT+ and combined CBT- groups in HYP-LS participants.

**HYP-HS**	**FLX+ *(FLX, CBT+FLX)***	**FLX- *(PBO, CBT)***	**Total**
	**(*n* = 74)**	**(*n* = 77)**	**(*n* = 151)**
**Observed**			
Responder (30% Improvement), *n (%)*	34 (45.9%)	19 (24.7%)	53 (35.0%)
Responder (40% Improvement), *n (%)*	25 (33.8%)	13 (16.9%)	38 (25.2%)
**Model Estimated Odds Ratio** ^a^			
Responder (30% Improvement), *OR (95% CI)*	2.70 (1.33, 5.48)	(ref)	
**HYP-LS**	**CBT+** ***(CBT, CBT+FLX)***	**CBT-** ***(PBO, FLX)***	**Total**
	**(*****n*** **= 17)**	**(*****n*** **= 25)**	**(*****n*** **= 42)**
**Observed**			
Responder (30% Improvement), *n (%)*	9 (52.9%)	6 (24.0%)	15 (35.7%)
Responder (40% Improvement), *n (%)*	8 (47.1%)	5 (20.0%)	13 (31.0%)
**Model Estimated Odds Ratio** ^a^			
Responder (30% Improvement), *OR (95% CI)*	8.03 (1.41, 45.67)	(ref)	

a *Adjusted for baseline baseline BDI score*.

In the analyses using the higher outcome threshold of a 40% improvement in symptoms to define responder status, the results were similar. Because 40% symptom improvement was achieved by a smaller number of participants (see Table 2, HYP-HS Observed), the power of the analyses was decreased. However, the estimated odds ratios were similar in magnitude and direction to those when 30% symptom improvement was used to define responder status.

In the exploratory moderator analyses within the HYP-HS group, no baseline moderators (dichotomized baseline Whiteley, H-YBOCS-M, and BDI) had a significant effect on association between FLX+/FLX- combined grouping and responder status, while adjusted for baseline BDI.

### Hypochondriacal Participants With Low Somatic Burden

Of the 42 HYP-LS participants, 15 (35.7%) individuals met the criteria for treatment response (see Table 2, HYP-LS Observed). The highest proportion of responders among HYP-LS participants was in the CBT+FLX treatment group (54.5%), followed by the CBT group (50.0%). Because of the small number of participants classified as HYP-LS, subsequent analyses were of very low power and focused on the magnitudes and direction of odds ratios and less on significance of the findings.

Based on the observed odds ratios of being a responder compared to PBO (see Table 2, HYP-LS Observed Odds Ratio), two new combined groups were formed. The odds ratios for being a responder in groups which received CBT (CBT and CBT+FLX) were similar in magnitude and direction and were combined into a new group labeled CBT+. The estimate of the effect of the FLX group did not differ in magnitude and direction from the PBO group, so these groups were combined into a new group labeled CBT- (Table 2, HYP-LS Model Estimates).

The new CBT+ combined group contained 9/17 (52.9%) participants who were treatment responders compared to 6/25 (24.0%) treatment responders in the CBT-combined group (see Figure 1 and Table 3, Observed). The model-estimated odds of being a responder significantly differed between the CBT+ and CBT- combined groups (*p* = 0.019); participants in the CBT+ combined group had 8.03 times (95% CI: 1.41, 45.67) the odds of being a responder compared to the CBT- combined group, while adjusting for baseline BDI scores (see Table 3, Model Estimated Odds Ratios). In the analyses using the 40% threshold of improvement to define responder status, the results were very similar (in magnitude and direction) to the results using the 30% threshold to define responder status.

### Sensitivity Analysis

Table 4 provides results when the definition of HYP-HS is based on different numbers of PHQ-15 items endorsed at the “Bothered a lot” level. Row 1 shows the results presented above, where HYP-HS is defined by endorsing at least 1 PHQ-15 item at the “Bothered a lot” level and HYP-LS is defined by no items endorsed “Bothered a lot.” This operational definition of HYP-HS and HYP-LS approximates the DSM-5 diagnoses of Somatic Symptom Disorder and Illness Anxiety Disorder. Subsequent rows show how the treatment results change as the definition of HYP-HS and HYP-LS change, based on the number of items endorsed at the “Bothered a lot” level. It is important to note that by altering the cutoff that defines the HYP-HS group, the makeup of HYP-LS group meaningfully changes.

**Table 4:** Sensitivity analysis: varying definition of HYP-HS and HYP-LS based on number of PHQ-15 items endorsed at the “Bothered a lot” level.

**Cutoff for # of PHQ-15**		**HYP-HS**	**HYP-LS**	**Effect of FLX+ in HYP-HS**	**Effect of CBT+ in HYP-LS**
**Items reported as “Bothered a lot”**		***n (%)***	***n (%)***	**Group *OR (95% CI)***	**Group *OR (95% CI)***
**HYP-HS**	**HYP-LS**				
≥1	0	151 (78.2%)	42 (21.8%)	**2.697 (1.327, 5.478)**	**8.029 (1.412, 45.667)**
≥2	≤1	109 (56.5%)	84 (43.5%)	**3.998 (1.676, 9.537)**	1.755 (0.681, 4.528)
≥3	≤2	74 (38.3%)	119 (61.7%)	**3.048 (1.097, 8.471)**	1.653 (0.739, 3.698)
≥4	≤3	53 (27.5%)	140 (72.5%)	1.935 (0.584, 6.407)	1.627 (0.785, 3.370)
≥5	≤4	31 (16.1%)	162 (83.9%)	0.781 (0.163, 3.749)	1.478 (0.759, 2.879)
≥6	≤5	17 (8.8%)	176 (91.2%)	2.315 (0.259, 20.679)	1.293 (0.685, 2.441)

*HYP-LS, Hypochondriasis-Low Somatic; HYP-HS, Hypochondriasis-High Somatic; FLX+ includes any treatment group in which fluoxetine was a component (FLX; FLX+CBT); CBT+ includes any treatment group in which CBT was a component (CBT; CBT+FLX). Highlighted effects are significant*.

Rows 2–6 in Table 4 also compare HYP-HS and HYP-LS, but the change in the number of PHQ-15 items for the criterion definition leads to a mixed HYP-LS group that no longer approximates a homogenous Illness Anxiety Disorder sample. For example, in the second row, half (*n* = 42) of the HYP-LS group consists of individuals who did not endorse a single PHQ-15 item as “Bothered a lot” (approximating IAD), while the other half (*n* = 42) endorsed exactly one item at the “Bothered a lot” level (approximating SSD).

Rows 2–3 of Table 4 show that the treatment effect of FLX+ in the HYP-HS group remains significant using a criterion of at least 2 or 3 PHQ endorsed items endorsed at the “Bothered a lot” level. Rows 2–6 demonstrate that the treatment effect of CBT+ in the HYP-LS group is no longer significant once this group contains individuals who have endorsed even one PHQ item at the “Bothered a lot” level.

### Adherence to Treatment

The proportion of participants assigned to CBT or CBT+FLX who completed all 6 core CBT sessions was 11/17 (64.7%) in the HYP-LS group and 54/88 (61.4%) in the HYP-HS group; this difference in adherence to CBT was not statistically significant (z = 0.260; *p* = 0.795).

The mean percentage of days fluoxetine was taken for those assigned to FLX or CBT+FLX groups was 68.0% in the HYP-LS group and 67.8% in the HYP-HS group; this difference in adherence to fluoxetine was not significantly different (t_df = 96_ = 0.024; *p* = 0.983). The mean final dose of fluoxetine was 29.6 mg for HYP-LS and 29.9 mg for HYP-HS; this difference in mean final dose was not significantly different (t_df = 96_ = 0.054; *p* = 0.957).

## Discussion

This exploratory study indicates that hypochondriacal participants with prominent somatic symptoms may be more likely to respond to fluoxetine than to CBT, while hypochondriacal participants without prominent somatic symptoms may be more likely to respond to CBT than to fluoxetine. This was demonstrated using both 30 and 40% thresholds to define responder status at the end of treatment compared to baseline.

Analyses within each of the hypochondriasis subgroups was illuminating as it demonstrated that joint therapy and a specific therapy, different for each subgroup, resulted in similar odds ratio (judging by size and direction). This enabled the combining of therapeutic modalities for a *post-hoc* exploratory analysis, as per Tukey and others (13, 14). Among the high somatic hypochondriacal individuals, joint therapy (45.2%) and fluoxetine therapy (46.9%) had similarly high responder rates, as compared to the CBT (28.3%) and placebo (19.4%) groups; the combined significant comparisons were 45.9% (any fluoxetine) vs. 24.7% (no fluoxetine) within the high somatic hypochondriacal group (Figure 1). Among the low somatic hypochondriacal individuals, joint therapy and CBT therapy had similarly high responder rates (54.5 and 50%), as compared to the fluoxetine and placebo groups (23 and 25.0%, respectively); the combined significant comparisons were 52.9% (any CBT) vs. 24.0% (no CBT) among the low somatic hypochondriacal individuals (Figure 1). These findings highlight the limited additional benefit of joint therapy within each of the somatic subgroups of hypochondriasis as well as the specific benefit of different therapeutic approaches within each of the subgroups.

In the main publication for this study, the primary agent of therapeutic change for the hypochondriacal sample was treatment with fluoxetine, as CBT contributed less benefit. The weakness of CBT was inconsistent with prior research; indeed this same CBT approach had previously been shown to be beneficial in a large controlled study conducted in primary care clinics (15) and several other studies (16, 17). Our new findings suggest that the CBT provided in this prior study actually had substantial clinical benefit, but that this benefit was primarily seen in the subgroup of hypochondriacal individuals without prominent somatic symptoms. The umbrella diagnosis of DSM-IV hypochondriasis may be too heterogeneous to enable optimal testing of the treatment efficacy of CBT among patients with illness anxiety.

Similar to an earlier study (2), we found that ~1 in 5 of individuals with DSM-IV hypochondriasis would likely meet criteria for the DSM-5 diagnosis of Illness Anxiety Disorder (our HYP-LS subgroup) and 4 in 5 would likely meet criteria for Somatic Symptom Disorder (our HYP-HS subgroup). Our HYP-LS and HYP-HS groups share similar symptoms, but the severity was greater in the HYP-HS group; the latter group had more severe anxiety, depression, and hypochondriasis, worse quality of life, and higher rates of major depression and panic disorder. These results are comparable to other reports in which individuals with illness anxiety and prominent somatic symptoms compared to illness anxiety with less somatic distress had significantly worse health anxiety, depression, functional status and higher rates of major depression and panic disorder (2, 18).

No face-to face studies and no comparative psychotherapy-medication studies have yet been published on the treatment of individuals with the DSM-5 diagnoses of IAD or SSD. To our knowledge, other than our prior study which did use a somatic symptom measure, the only other psychotherapy vs. medication randomized controlled trial of hypochondriasis used DSM-III-R criteria and did not assess somatic symptoms, thereby preventing an analysis of somatic subtypes as conducted in this paper (19). Two internet studies of health anxiety have recruited patients who meet the DSM-5 criteria for IAD and SSD to assess the impact of exposure-based CBT. One study reported that 12 sessions of iCBT showed greater benefit than a wait-list control at 3 months in a combined group of SSD (*n* = 114) and IAD (*n* = 18) participants; while this study demonstrated that exposure-based iCBT was effective in the combined group of those with SSD and IAD, the small number of IAD participants prevented a comparison of the efficacy of iCBT between these two diagnostic groups (20). A smaller study also using a combined group of individuals with IAD (*n* = 35) and SSD (*n* = 29) compared 6 sessions of iCBT to psychoeducation about anxiety reduction; in this study, a secondary subgroup analysis suggested that iCBT led to a comparably greater reduction in health anxiety than did psychoeducation group within each of these two diagnostic groups, but the sample size was too small to assess non-inferiority (21). It is unclear from these studies whether it was the components of iCBT itself that were beneficial or simply that a more structured psychological treatment was compared to a wait-list control or minimal educational treatment. It is not possible to compare these studies to the present study given the many differences in study design. Nevertheless, these studies are promising in suggesting that health anxious patients with either SSD or IAD may well benefit from exposure-based CBT. Given the lack of a medication group comparator, these iCBT studies do not address the issue of whether pharmacotherapy might have provided even greater benefit in the SSD patients.

Our study used clinically relevant cutoffs for somatic symptoms to define the HYP-HS and HYP-LS groups, as these cutoffs correspond to the somatic symptom criteria used in DSM-5 for Illness Anxiety Disorder and Somatic Symptom Disorder. The sensitivity analysis examined whether the results of our study would vary if different cutoffs for somatic symptoms were selected to define HYP-HS and HYP-LS. While the results for fluoxetine did not change meaningfully based on number of somatic symptoms endorsed in the HYP-HS group, this analysis also demonstrated that the preferential benefit of CBT in the HYP-LS group was present only when the definition of HYP-LS excluded individuals with one or more prominent somatic symptoms. These results lend some support to the distinction made in DSM-5 between IAD and SSD.

Prior studies of CBT for hypochondriasis/health anxiety revealed that those with concurrent depression were less likely to benefit from CBT than those with lower rates of concurrent depression (17). This may be one explanation why the HYP-HS patients in our study responded poorly to CBT while the HYP-LS patients responded well, as the former group had greater prominent depression. Another consideration is that the manualized CBT was designed to treat hypochondriasis—not depression. It is conceivable that patients with HYP-HS may benefit from an expanded CBT protocol that incorporates aspects that treat both depression and health anxiety. Additionally, a greater number of CBT sessions and ones which incorporate exposure interventions may increase efficacy. A related study is relevant here. A secondary analysis of a randomized controlled trial study of CBT for hypochondriasis conducted in multiple primary care settings assessed whether the presence of chronic back pain had an impact on responsiveness to treatment; the analysis revealed that CBT was not effective in reducing hypochondriasis among hypochondriacal participants with chronic lower back pain while it was effective among hypochondriacal individuals without chronic low back pain (22). Results from this adult hypochondriasis back pain study are consistent with the findings presented in this paper.

Fluoxetine appears to have out-performed CBT in the HYP-HS group. Part of the explanation may be that fluoxetine is a broad-acting pharmacotherapy agent with demonstrated efficacy in reducing depression, anxiety, hypochondriasis, and obsessive-compulsive disorder, whereas the manualized CBT is focused primarily on health anxiety and not on the other accompanying psychopathology associated with Somatic Symptom Disorder. Another possible explanation is that pharmacotherapy for depression is more effective as severity of depression increases (23).

Could differences in treatment adherence account for these differences in response to CBT and fluoxetine therapy in this study? In the iCBT study of health anxiety (21), more severely ill patients had a higher drop-out rate. In the present study, the proportion of patients who completed all 6 core CBT sessions was comparable for HYP-LS and HYP-HS; this suggests that difference in rates of adherence to CBT would not account for the different responder patterns. There were also no differences between the HYP-LS and the HYP-HS groups in the adherence to fluoxetine treatment.

A major strength of this exploratory study is the use of a unique database from the only clinical trial to our knowledge that has ever compared joint therapy, individual psychotherapy and pharmacotherapy, and placebo among individuals with hypochondriasis. Of particular significance is that this large two-site clinical trial was conducted before the formulation of the DSM-5 nosology; this meant that clinicians were not focused on somatic symptoms but treated hypochondriasis using standard CBT and pharmacotherapy approaches, thus reducing bias that might have emerged had hypotheses about differential treatment responsiveness been present. An additional strength is that, based on results from the original study, we used a more rigorous threshold of 30% to define responders rather than the 25% originally chosen; this a priori decision was made to enhance the detection of differences in responder rates between active and placebo treatments (3). Finally, adherence to treatment was comparable for CBT and for fluoxetine within both the HYP-LS and HYP-HS groups.

A limitation of this study is that the subsample of patients with HYP-LS was relatively small for the 4-treatment group analysis, resulting in potentially unreliable odds ratios with wide confidence intervals and low power. However, the subsequent exploratory analysis comparing those who received any CBT to those who had not received CBT provided more reliable evidence with higher power, demonstrating a significantly different responder rate favoring CBT in the HYP-LS sample (OR = 8.03; 95% CI: 1.41, 45.67). Our original study was not designed for this exploratory analysis. Our findings, though exploratory and based on a small sample size, were obtained through valid statistical methods. These results may be useful as guidance for future studies as these can only suggest potential differential treatment effects which need to be confirmed using larger and more specifically designed studies.

Secondly, our findings cannot be generalized directly to DSM-5 IAD and SSD because we compared DSM-IV hypochondriacal individuals with and without prominent somatic distress, not individuals meeting DSM-5 criteria for IAD and SSD. The diagnosis of SSD encompasses a broader group of individuals including, for example, those who previously would have received the diagnosis of pain disorder; our findings however would be generalizable to ICD-11 hypochondriasis as the criteria are similar (but not identical) to DSM-IV (24). Third, while these results indicate that CBT was not effective for hypochondriasis with high somatic burden, the CBT treatment approach did not include exposure therapy—a specific component that may be needed for the more severely ill patients with hypochondriasis and prominent somatic symptoms.

In conclusion, this study indicates that patients with DSM-IV hypochondriasis with high or low somatic distress may respond differentially to CBT and fluoxetine. The results from this exploratory study suggest a direction for future, more definitive studies. To our knowledge, this is the first report exploring the relative benefits of pharmacotherapy vs. psychotherapy in a head-head comparison in hypochondriacal individuals with and without prominent somatic symptoms. Systematic replication of these findings in larger studies is needed to confirm that the presence or absence of prominent somatic symptoms is a useful guide for clinicians in selection of treatment for individuals with hypochondriasis.

## Data Availability Statement

The data analyzed in this study is subject to the following licenses/restrictions: the original consents for this study were written in 2004 – i.e., before consent forms routinely contained information about data sharing in public repositories. However, this dataset is available on request. The raw data supporting the conclusions of this article will be made available by the authors, without undue reservation. Requests to access these datasets should be directed to Brian A. Fallon, baf1@cumc.columbia.edu.

## Ethics Statement

The studies involving human participants were reviewed and approved by the New York State Psychiatric Institute IRB and by the Partners Healthcare IRB. The patients/participants provided their written informed consent to participate in this study.

## Author Contributions

BF, CB, MP, DA, and AB contributed to the design and implementation of the research, to the analysis of the results, and to the writing of the manuscript. All authors contributed to the article and approved the submitted version.

## Funding

This work was supported by NIMH grants to BF (RO1 MH071456) and AB (RO1 MH071688).

## Conflict of Interest

The authors declare that the research was conducted in the absence of any commercial or financial relationships that could be construed as a potential conflict of interest.

## Publisher's Note

All claims expressed in this article are solely those of the authors and do not necessarily represent those of their affiliated organizations, or those of the publisher, the editors and the reviewers. Any product that may be evaluated in this article, or claim that may be made by its manufacturer, is not guaranteed or endorsed by the publisher.
